# Prevalence and Associated Factors of Hypertension Among School-Going Children in Hyderabad City, India: A Cross-Sectional Study

**DOI:** 10.7759/cureus.117266

**Published:** 2026-09-30

**Authors:** Prajakta Supe, Suneel Pooboni, Sandya P, Mohd Saeed Siddiqui

**Affiliations:** 1 Pediatrics, MGM Medical College and Hospital, MGM Institute of Health Sciences, Aurangabad, IND; 2 Pediatrics, Pediatric Intensive Xmcare, Dubai, ARE; 3 Pediatrics, Krishna Institute of Medical Sciences, Secunderabad, IND

**Keywords:** body mass index (bmi), childhood hypertension, childhood obesity, india, prehypertension, school health services

## Abstract

Background

Childhood hypertension is an increasingly important public health concern because elevated blood pressure during childhood predisposes individuals to hypertension and cardiovascular disease in adulthood. Although obesity is recognised as the principal modifiable risk factor, regional data from India remain heterogeneous. This study aimed to estimate the prevalence of hypertension and prehypertension among school-going children in Hyderabad and determine its association with body mass index (BMI) and other correlates of elevated blood pressure.

Methods

We report this school-based cross-sectional study. A total of 1,212 children aged 7-15 years from two government and two private schools in Hyderabad were studied between August 2014 and May 2015. Anthropometric and blood pressure measurements were obtained using standardised procedures. Blood pressure was measured three times during the same visit, at 0, 30, and 60 minutes, and the readings were averaged. Hypertension and prehypertension were defined by age-, sex-, and height-specific percentiles from the Fourth Report of the National High Blood Pressure Education Program Working Group. Associations were examined using chi-square tests, and independent factors with adjusted odds ratios (aOR) were identified by multivariable binary logistic regression.

Results

Mean systolic blood pressure (SBP) and diastolic blood pressure (DBP) rose progressively across BP categories (normal BP, prehypertension, hypertension: 100.9-126.1 mmHg and 62.8-75.3 mmHg, respectively; p < 0.001 for both), and both parameters correlated significantly with age and, more strongly, with BMI (ρ = 0.50-0.54 and ρ = 0.31-0.35, respectively; p < 0.001). The overall prevalence of hypertension was 6.68% (95% CI: 5.41-8.23%) and prehypertension 8.09% (95% CI: 6.68-9.76%). Among 967 normal-weight, 130 overweight, and 115 obese children, hypertension prevalence was 2.1% (95% CI: 1.3-3.2%), 12.3% (95% CI: 7.5-18.7%), and 39.1% (95% CI: 29.2-49.9%), respectively (Pearson chi-square = 437, p < 0.001). In multivariable analysis, obesity (aOR: 8.60, 95% CI: 3.84-19.26) and overweight (aOR: 3.01, 95% CI: 1.33-6.79) remained strongly associated with hypertension, along with parental history of hypertension (aOR: 2.14, 95% CI: 1.12-4.09), mixed dietary pattern (aOR: 1.97, 95% CI: 1.07-3.61) and male sex (aOR: 1.93, 95% CI: 1.11-3.36). Model discrimination was good (AUC: 0.886, 95% CI: 0.84-0.93).

Conclusions

In this Hyderabad school-based study, prehypertension and hypertension were common and showed a strong, graded association with BMI category. Blood pressure showed a significant, graded rise across hypertension categories that was more strongly linked to BMI than to age. While cross-sectional design precludes causal inference, findings align with national pooled estimates. It implies that surveillance and school-based healthy weight initiatives with targeted blood pressure screening using repeated measurements should continue. Multi-centric, longitudinal studies using the latest criteria and ambulatory blood pressure monitoring are needed to confirm causality and secular trends.

## Introduction

Hypertension in childhood often persists into adulthood and contributes to cardiovascular morbidity. Population studies show measurable hypertension and prehypertension among school-aged children, with prevalence rising from 2% in normal-weight children to 11% in obese children, and overweight and ethnicity as key correlates [1]. Secular trends indicate no consistent decline in childhood blood pressure (BP); mean systolic blood pressure (SBP) and diastolic BP (DBP) increased by 1.4 and 3.3 mmHg, respectively, between 1988-1994 and 1999-2000 [2]. In India, pediatric hypertension has received less attention than adult hypertension, despite recommendations emphasizing early detection to prevent long-term complications [3].

The rising burden parallels global increases in childhood overweight. South Asian children exhibit higher body mass index (BMI)-adjusted BP than white children in the United States (overall high BP of 12.2%, boys of 15.8%, girls of 8.7%), suggesting heightened susceptibility linked to genetic, dietary, and environmental factors [4]. India is undergoing rapid nutritional transition with rising urban childhood obesity [5]. Asian Indian children also show early insulin resistance and cardiometabolic abnormalities predisposing to hypertension [6]. Excess adiposity raises BP via sympathetic activation, insulin resistance, altered renal sodium handling, and vascular dysfunction, making obesity the principal modifiable determinant [7].

Reported prevalence in India varies widely due to differences in populations, methods, and diagnostic criteria. Studies from Ludhiana and Shimla reported urban-rural differences [8,9], while Surat reported 6.48% prevalence with obesity and family history as key associated factors [10]. Kerala data showed progressive BP increase across BMI categories [11], with similar gradients in Odisha adolescents [12]. A recent systematic review from India reported a pooled hypertension prevalence of 7% (95% CI: 6%-8%) and prehypertension of 10% (95% CI: 8%-13%), noting a rising trend after 2005, higher rates in urban than rural children, and a markedly higher prevalence among obese children (29%) compared with normal-weight children (7%) [13]. A global systematic review similarly estimated sustained pediatric hypertension at 3.89% (95% CI: 3.27%-4.62%) and occasional hypertension at 11.85% (95% CI: 10.98%-12.75%), with substantially higher prevalence among overweight and obese children (6.79% and 16.35%, respectively) than normal-weight children (2.57%) [14].

Reported childhood hypertension prevalence in India varies, reflecting differing age groups, reference standards, cuff selection, and repeat-visit confirmation, limiting local applicability. Although BP screening is included in school health programmes, it is rarely performed in asymptomatic children, so elevated readings remain undetected. Hyderabad's rapid urbanization, nutrition transition, and mixed government-private schools make it a suitable setting for locally relevant estimates, while the interrelation of adiposity, diet, socioeconomic status, and family history warrants multivariable analysis to identify adjusted risk factors.

The primary objective of the study was to estimate the prevalence of hypertension and pre-hypertension among children aged 7-15 years in Hyderabad as per the Fourth Report of the National High Blood Pressure Education Program Working Group. The secondary objectives were to (1) evaluate the BMI-hypertension association and (2) identify independent associated factors.

## Materials and methods

Study design and setting

This was a school-based cross-sectional study conducted between August 2014 and May 2015 in western Hyderabad, Telangana. Approval was obtained from the institutional ethics committee (approval no. KFRC/EC/2014/33-05, approved July 23, 2014). Written parental consent and verbal assent from eligible children were obtained. 

Study population and sampling

All apparently healthy children aged 7-15 years present on survey days were eligible. Children with known chronic illness, congenital anomalies, disability, or chronic steroid use were excluded. The sampling frame comprised all eligible schools in Hyderabad and was stratified into (i) government and government-aided schools and (ii) private unaided schools. Two schools from each stratum were selected using convenience sampling. Within each selected school, eligible children from the prespecified age/grade groups were sampled until the required number was achieved. The required sample size under simple random sampling was 626 children, calculated assuming an expected prevalence of 7% [10], an absolute precision of 2 percentage points, and a 95% confidence level. This was inflated by a design effect of approximately 2.0 to account for clustering of children within schools, giving a target sample of approximately 1,251 children. Of 1,240 approached, 1,212 children were enrolled.

Data collection and measurements

The study proforma was developed and pretested to collect required relevant data. Each child was given a proforma to be filled at home with the help of parents. Parents/guardians were also provided with a description of the project and informed consent forms. A mixed diet was defined as any non-vegetarian intake ≥ once/week, and a vegetarian diet as no meat/fish/egg. Junk food (fried snacks, sugar-sweetened beverages, bakery, fast-food) frequency in the past seven days was recorded as <=1, 2, 3, ≥ 4/week. Low physical activity was defined as self-reported moderate-to-vigorous activity <30 min/day. Socioeconomic status was assessed as per the modified Kuppuswamy scale [15] using 2007 income cut-offs. A history of parental hypertension was self-reported. Weight was measured to 0.1 kg (calibrated electronic scale, light clothes, no shoes) and height to 0.1 cm (portable stadiometer, Frankfurt plane). BMI was calculated as kg/m² and categorized as per Indian Academy of Pediatrics (IAP) 2007 charts as normal (<85th percentile), overweight (85th-<95th percentile), and obese (≥95th percentile) [16].

Blood pressure measurement

Blood pressure was measured in accordance with the recommendations of the Fourth Report on the Diagnosis, Evaluation, and Treatment of High Blood Pressure in Children and Adolescents [17]. Prior to data collection, permission was obtained from the head of each participating institution, and the procedure was explained to each child in age-appropriate terms, with reassurance that it was neither painful nor harmful; measurements were taken under simulated "basal" or "near-basal" conditions, with efforts made to minimize anxiety, fear, crying, laughing, recent physical activity, and other factors known to transiently affect BP. After a five-minute seated rest, BP was recorded by trained observers in the right arm using the auscultatory method with a calibrated mercury sphygmomanometer (Diamond); children sat with the back supported, feet flat on the floor, and the arm resting at heart level on a table, with the cuff tied midway between the acromion and olecranon processes. Cuff size was selected for each child based on measured mid-upper arm circumference (MUAC) rather than age, from a standardized set of cuffs (child, small adult, adult), such that the inflatable bladder width approximated 40% of MUAC and the bladder length covered 80-100% of arm circumference. The cuff was inflated until the distal arterial pulse was no longer palpable and then deflated at a rate of approximately 2-3 mm Hg per second; SBP was recorded at the first Korotkoff sound (phase I) and DBP at the disappearance of Korotkoff sounds (phase V). Three readings were obtained on the same day at 0, 30, and 60 minutes, and their average, rounded to the nearest even integer, was used for analysis. Hypertension was defined as mean systolic and/or diastolic BP ≥95th percentile, and prehypertension as 90th to <95th percentile, for age, sex, and height per the Fourth Report tables [17]. As BP was assessed on a single occasion, the study estimates single-visit elevated BP/hypertension according to these criteria rather than confirmed persistent hypertension.

Children classified as prehypertensive or hypertensive were referred to a tertiary care hospital for further evaluation. There, they were examined by a pediatrician, underwent repeat blood pressure measurement, and had a baseline workup comprising renal function tests and electrocardiography, with consultation from a cardiologist and/or nephrologist as indicated. Further management was instituted according to the specialist's recommendations.

Statistical analysis

Data were entered in Microsoft Excel (Microsoft® Corp., Redmond, WA) and analysed using Jamovi software (version 2.4.1). Normality was assessed using the Shapiro-Wilk test and Q-Q plots. Continuous variables were summarized as mean ± SD, while categorical variables were expressed as frequencies and percentages with 95% confidence intervals. Group comparisons were performed using Pearson's chi-square test or Fisher's exact test for categorical variables and one-way ANOVA or the Kruskal-Wallis test for continuous variables.

Hypertension prevalence was reported overall and by BMI category. Binary logistic regression was used to identify factors associated with hypertension. Variables with p < 0.20 in univariable analysis and clinically relevant covariates were included in the multivariable model. Model performance was assessed using likelihood-ratio chi-square, McFadden's pseudo-R², AIC, and AUC with 95% confidence intervals. A p-value < 0.05 was considered statistically significant.

## Results

A total of 1,212 school-going children aged 7-15 years were analysed (mean age: 11.1 ± 2.5 years). Sex distribution was comparable across BP categories (p = 0.152). School type and socioeconomic status were significantly associated with BP status (both p < 0.001), with higher proportions of children from private schools and lower socioeconomic groups in the pre-hypertension and hypertension categories. Lifestyle factors also differed significantly across BP groups. Lower physical activity (< 30 minutes/day), mixed diet, and frequent junk-food consumption (≥4 times/week) were increasingly common among children with elevated BP (all p < 0.001). BMI showed the strongest association with BP status; obesity was present in 41 (4.0%) of children with normal BP, 29 (29.6%) with pre-hypertension, and 45 (55.6%) with hypertension (p < 0.001). Older age was associated with higher BP categories, with the proportion of children aged 14-15 years increasing from 208 (20.1%) in the normal-BP group to 30 (37.0%) in the hypertension group. Mean age also increased significantly across BP categories (10.9 vs. 12.3 years; F = 26.24, p < 0.001). Table 1 shows participant characteristics across BP categories.

**Table 1:** Participant characteristics according to blood pressure status Data are mean (SD) or n (%). BP: blood pressure; SES: socioeconomic status; BMI: body mass index; SD: standard deviation; χ²: chi-square

Characteristic	Category	Normal BP (N=1033)	Prehypertension (N=98)	Hypertension (N=81)	Total (N=1212)	df	X^2^ Statistic	p value
Sex	Female	498.0 (48.2%)	46.0 (46.9%)	30.0 (37.0%)	574.0 (47.4%)	2	3.77	0.1522
	Male	535.0 (51.8%)	52.0 (53.1%)	51.0 (63.0%)	638.0 (52.6%)			
School	Government	559.0 (54.1%)	40.0 (40.8%)	28.0 (34.6%)	627.0 (51.7%)	2	16.58	< 0.001
	Private	474.0 (45.9%)	58.0 (59.2%)	53.0 (65.4%)	585.0 (48.3%)			
SES	Lower	212.0 (20.5%)	30.0 (30.6%)	36.0 (44.4%)	278.0 (22.9%)	4	29.69	< 0.001
	Middle	594.0 (57.5%)	47.0 (48.0%)	28.0 (34.6%)	669.0 (55.2%)			
	Upper	227.0 (22.0%)	21.0 (21.4%)	17.0 (21.0%)	265.0 (21.9%)			
Physical Activity	< 30 min/day	147.0 (14.2%)	39.0 (39.8%)	45.0 (55.6%)	231.0 (19.1%)	2	112.87	< 0.001
	≥ 30 min/day	886.0 (85.8%)	59.0 (60.2%)	36.0 (44.4%)	981.0 (80.9%)			
Diet	Mixed	376.0 (36.4%)	62.0 (63.3%)	61.0 (75.3%)	499.0 (41.2%)	2	68.44	< 0.001
	Vegetarian	657.0 (63.6%)	36.0 (36.7%)	20.0 (24.7%)	713.0 (58.8%)			
Junk Food Frequency	1 x/week	316.0 (30.6%)	9.0 (9.2%)	12.0 (14.8%)	337.0 (27.8%)	6	198.08	< 0.001
	2 x/week	502.0 (48.6%)	41.0 (41.8%)	11.0 (13.6%)	554.0 (45.7%)			
	3 x/week	159.0 (15.4%)	28.0 (28.6%)	21.0 (25.9%)	208.0 (17.2%)			
	≥ 4x/week	56.0 (5.4%)	20.0 (20.4%)	37.0 (45.7%)	113.0 (9.3%)			
BMI	Normal (< 85th)	919.0 (89.0%)	28.0 (28.6%)	20.0 (24.7%)	967.0 (79.8%)	4	437.49	< 0.001
	Overweight (85-95th)	73.0 (7.1%)	41.0 (41.8%)	16.0 (19.8%)	130.0 (10.7%)			
	Obese (> 95th)	41.0 (4.0%)	29.0 (29.6%)	45.0 (55.6%)	115.0 (9.5%)			
Age Group	7-9 Years	347.0 (33.6%)	16.0 (16.3%)	12.0 (14.8%)	375.0 (30.9%)	6	54.65	< 0.001
	10-11 Years	250.0 (24.2%)	11.0 (11.2%)	13.0 (16.0%)	274.0 (22.6%)			
	12-13 Years	228.0 (22.1%)	31.0 (31.6%)	26.0 (32.1%)	285.0 (23.5%)			
	14-15 Years	208.0 (20.1%)	40.0 (40.8%)	30.0 (37.0%)	278.0 (22.9%)			

Hypertension prevalence increased markedly across BMI categories, affecting 20 (2.1%) of children with normal BMI, 16 (12.3%) of overweight children, and 45 (39.1%) of obese children. Pre-hypertension showed a similar pattern, occurring in 28 (2.9%), 41 (31.5%), and 29 (25.2%) of these groups, respectively. Most participants had normal BMI (79.8%; n = 967), while 10.7% (n = 130) were overweight and 9.5% (n = 115) were obese. Obese hypertensive children constituted 45 (3.7%) of the total cohort, compared with 16 (1.3%) who were both overweight and hypertensive. BMI category was strongly associated with blood pressure status (χ² = 437, df = 4, p < 0.001) (Figure 1).

**Figure 1:**
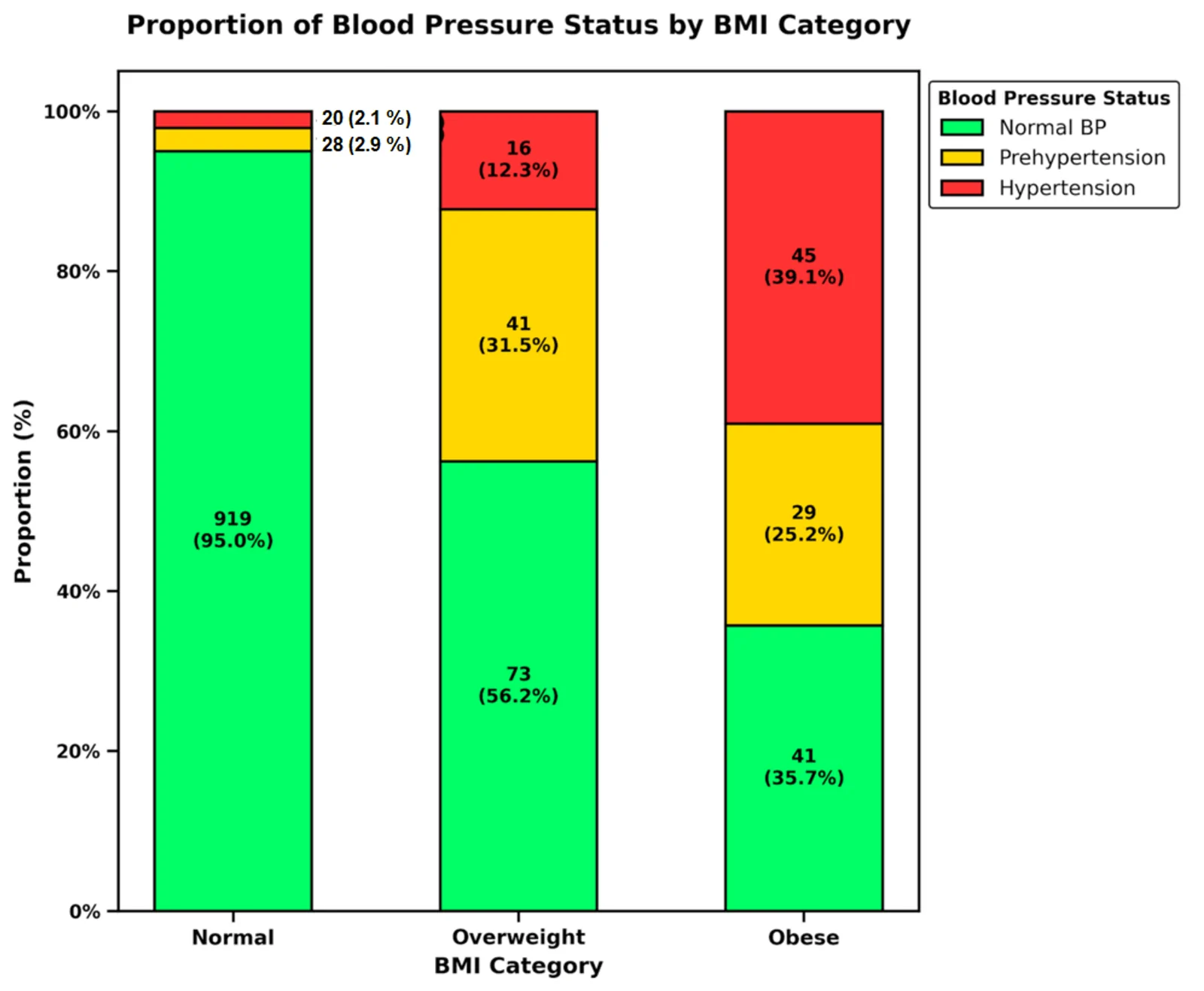
Proportion of blood pressure status across BMI category BMI: body mass index

Among the 1,212 children studied, mean SBP and DBP were 104.2 ± 10.2 mmHg and 64.1 ± 6.6 mmHg overall, respectively. Values rose progressively across hypertension categories: SBP was 100.9 ± 6.8, 120.2 ± 3.8, and 126.1 ± 6.0 mmHg, and DBP was 62.8 ± 5.4, 68.9 ± 6.1, and 75.3 ± 7.4 mmHg in the normal BP, prehypertension, and hypertension groups, respectively. This gradient was highly significant on Kruskal-Wallis testing for both SBP (H(2) = 444.4, p < 0.001, ε² = 0.37) and DBP (H(2) = 223.6, p < 0.001, ε² = 0.18), reflecting large effect sizes. Pairwise comparisons (Dwass-Steel-Critchlow-Fligner test) showed that normal BP differed significantly from both prehypertension and hypertension for SBP and DBP (all p < 0.001) and that prehypertension differed significantly from hypertension for both SBP and DBP (both p < 0.001), indicating that all three BP categories were mutually distinguishable on both parameters. SBP and DBP also correlated significantly with age (ρ = 0.50 and ρ = 0.31) and BMI (ρ = 0.54 and ρ = 0.35; all p < 0.001), with BMI showing the stronger association for both parameters, and children with prehypertension or hypertension clustering toward higher age and BMI values (Figures 2, 3).

**Figure 2:**
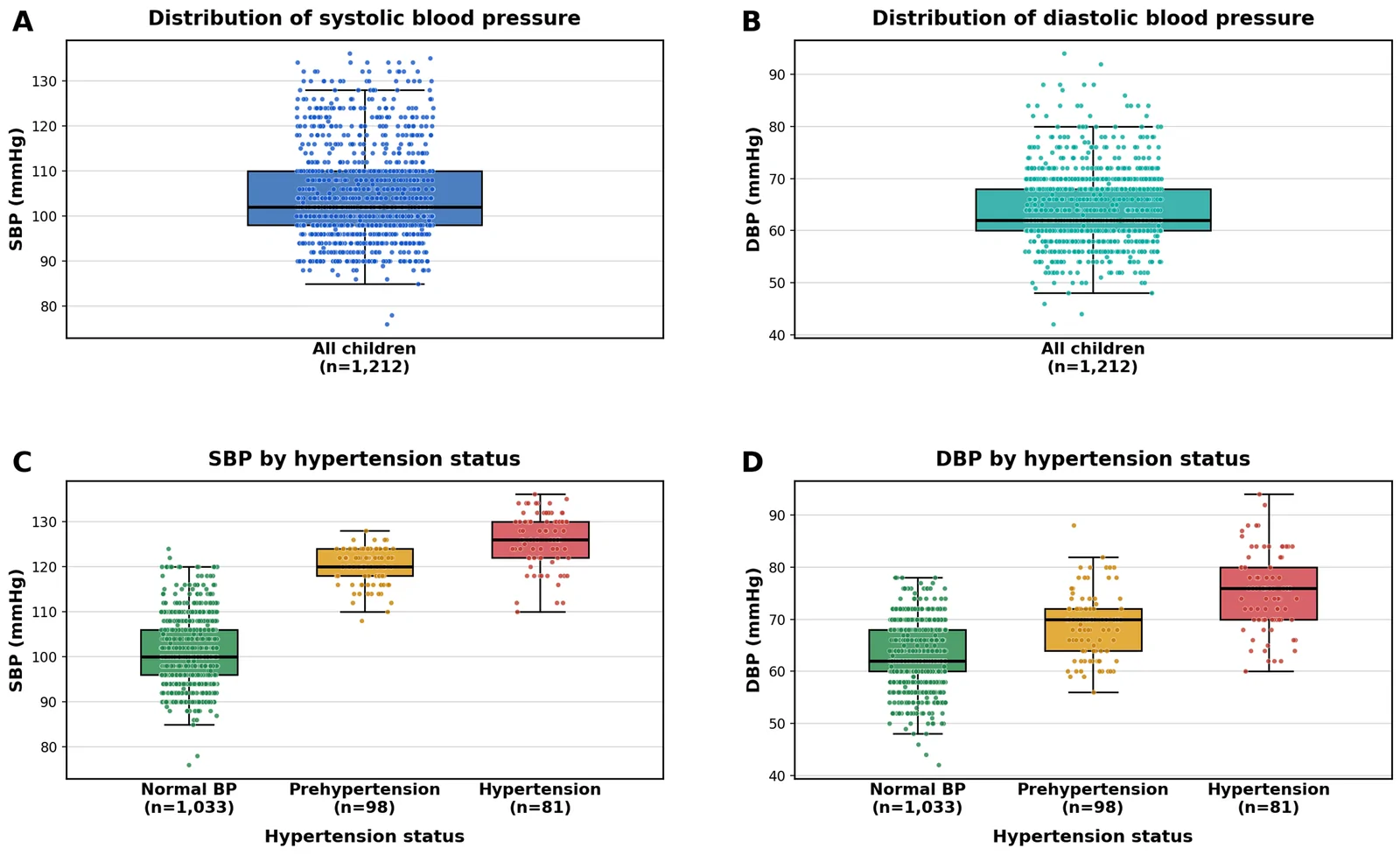
Distribution of systolic and diastolic blood pressure Distribution of systolic blood pressure (SBP) and diastolic blood pressure (DBP) in the cohort (n = 1,212). (A, B) Box-and-jitter plots of SBP and DBP for all children; boxes show median and interquartile range, whiskers show 1.5×IQR, and points represent individual measurements. (C, D) SBP and DBP stratified by hypertension status (normal BP, n = 1,033; prehypertension, n = 98; hypertension, n = 81). Group differences were tested with the Kruskal-Wallis test, with pairwise comparisons by the Dwass-Steel-Critchlow-Fligner test.

**Figure 3:**
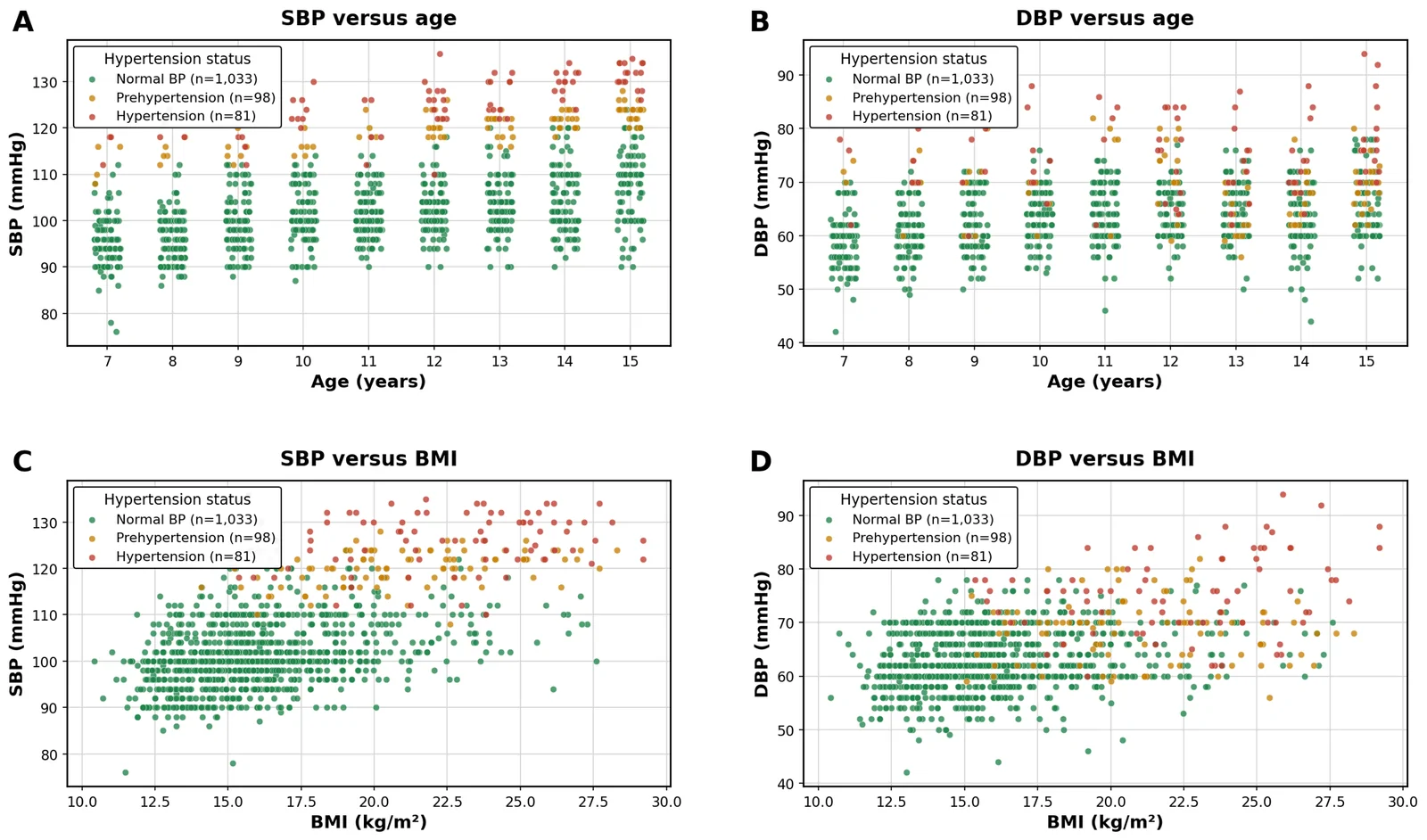
Scatter plots of systolic and diastolic blood pressure against age and BMI Relationship of systolic blood pressure (SBP) and diastolic blood pressure (DBP) with age and body mass index (BMI). (A, B) SBP and DBP versus age in years; (C, D) SBP and DBP versus BMI. Points are colored by hypertension status. Correlations were tested using Spearman rho (ρ).

In univariate logistic regression, obesity showed the strongest association with hypertension (OR = 30.44, 95% CI: 17.04-54.36, p < 0.001), followed by junk food intake ≥4 times/week (OR = 13.19, 95% CI: 6.56-26.49, p < 0.001) and family history of hypertension (OR = 7.89, 95% CI: 4.70-13.23, p < 0.001). Junk food consumption demonstrated a dose-response relationship, with significantly increased odds at ≥3 times/week. Overweight status, low physical activity, mixed diet, private schooling, and lower socioeconomic status were also significantly associated with hypertension (all p ≤ 0.012), whereas male sex did not reach statistical significance (p = 0.056) (Table 2).

**Table 2:** Univariable logistic regression analysis of factors associated with hypertension Reference categories: normal BMI, physical activity >=30 min/day, female sex, government school, no parental history of hypertension, middle/upper socioeconomic status, and vegetarian diet. OR: odds ratio; CI: confidence interval; BMI: body mass index; SES: socio economic status; H/O: history of; SE: standardized estimate; Z: statistic value

Predictor	Estimate	SE	Z	Crude OR	95% CI	p value
Overweight	1.89	0.35	5.42	6.65	3.35-13.19	< 0.001
Obese	3.42	0.3	11.5	30.44	17.04-54.36	< 0.001
Physical activity < 30 min	1.85	0.24	7.78	6.35	3.99-10.12	< 0.001
Male	0.455	0.24	1.91	1.58	0.99-2.51	0.056
Private School	0.759	0.24	3.15	2.14	1.33-3.43	0.002
Family H/O Hypertension	2.07	0.26	7.83	7.89	4.70-13.23	< 0.001
Lower SES	0.775	0.31	2.52	2.17	1.19-3.97	0.012
Mixed Diet	1.57	0.27	5.94	4.83	2.87-8.11	< 0.001
Junk Food 2 x/week	-0.6	0.42	-1.42	0.55	0.24-1.26	0.156
Junk Food 3 x/week	1.112	0.37	2.98	3.04	1.46-6.32	0.003
Junk Food ≥ 4x/week	2.579	0.36	7.25	13.18	6.56-26.49	< 0.001

In multivariable analysis, obesity remained the strongest independent predictor of hypertension (aOR = 8.60, 95% CI: 3.84-19.26, p < 0.001), followed by overweight status (aOR = 3.01, 95% CI: 1.33-6.79, p = 0.008). Family history of hypertension, male sex, and mixed diet also remained significant predictors. Associations with physical activity, school type, socioeconomic status, and junk food intake were attenuated after adjustment. The final model was significant (χ² = 193, df = 12, p < 0.001), explained 32.5% of the variance (McFadden's R² = 0.325; AIC = 428), and demonstrated good discrimination (AUC = 0.886, 95% CI: 0.84-0.93) (Table 3).

**Table 3:** Multivariable logistic regression analysis of factors independently associated with hypertension Reference categories: normal BMI, physical activity >=30 min/day, female sex, government school, no parental history of hypertension, middle/upper socioeconomic status, vegetarian diet, and junk food consumption =<1 times/week. OR: odds ratio; CI: confidence interval; AUC: area under the receiver operating characteristic curve; BMI: body mass index; SES: socio economic status; H/O: history of; SE: standardized estimate; Z: statistic value

Predictor	Estimate	SE	Z	p value	adjusted OR	95% CI	p value
Overweight	1.1007	0.42	2.65	0.008	3.01	1.33-6.79	0.008
Obese	2.1522	0.41	5.23	< .001	8.60	3.84-19.26	< 0.001
Physical activity < 30 min	0.3467	0.31	1.12	0.262	1.41	0.77-2.59	0.262
Male	0.6587	0.28	2.34	0.019	1.93	1.11-3.36	0.019
Private School	0.412	0.29	1.42	0.156	1.51	0.85-2.67	0.156
Family H/O Hypertension	0.7628	0.33	2.31	0.021	2.14	1.12-4.09	0.021
Lower SES	0.5783	0.37	1.55	0.122	1.78	0.86-3.71	0.122
Mixed Diet	0.6772	0.31	2.19	0.029	1.97	1.07-3.61	0.029
Junk Food ≥ 4/week	0.6094	0.45	1.36	0.176	1.84	0.76-4.44	0.176

## Discussion

In this school-based cross-sectional study of 1,212 children aged 7-15 years surveyed in 2014-2015 in Hyderabad, the prevalence of hypertension was 6.68%, and 8.09% for prehypertension. BMI was the strongest factor associated with hypertension, with a clear graded increase across normal-weight, overweight, and obese categories. Parental history of hypertension, mixed dietary pattern, and male sex also remained associated after multivariable adjustment. In contrast, school type, physical activity, socioeconomic status, and frequent junk food consumption were not independently associated after adjustment. These findings reinforce the dominant contribution of excess adiposity to childhood hypertension while indicating that familial and selected demographic and dietary characteristics may contribute additional risk.

The prevalence observed closely corresponds with contemporary evidence from India. A systematic review and meta-analysis of 64 Indian studies reported pooled childhood hypertension prevalence of 7% (95% CI: 6-8%) [13]. Daniel et al. reported a pooled prevalence of 7.6% among Indian adolescents but highlighted substantial heterogeneity due to differences in sampling, BP measurement protocols, and diagnostic criteria [18]. Close agreement suggests that, although conducted in a single metropolitan region and a decade ago, present results remain similar to broader epidemiology, though secular trends toward higher obesity may mean current prevalence is higher.

Our prevalence is higher than pooled global estimates. Song et al. estimated worldwide prevalence of 4.0% using studies based predominantly on repeated BP measurements [19], while Ruan et al. reported 3.89% for sustained hypertension among individuals younger than 19 years [14]. Zhou et al. demonstrated that although childhood hypertension has increased globally over the past two decades, prevalence remains lower in most regions than in South Asia [20]. These findings are consistent with the well-recognized cardiometabolic susceptibility of South Asian populations.

In our study, the prevalence of hypertension rose steadily with age, from 3.2% among 7-9-year-olds to 10.8% at 14-15 years (with prehypertension rising correspondingly from 4.3% to 14.4%), and age group was significantly associated with hypertension status. This graded increase closely parallels Meena et al., who reported hypertension prevalence rising from 2% at 5 years to 9% at 16 years, and is broadly consistent with Song et al., who described prevalence increasing from 4.32% at 6 years to a peak of 7.89% at 14 years before declining in late adolescence [13,19]. Our peak prevalence in the 14-15-year group (10.8%) was somewhat higher than the peaks reported in both studies, which may reflect differences in study population, measurement protocol, or the specific age bands compared. As our oldest category was 14-15 years, we could not determine whether prevalence declines beyond this age as reported by Song et al., and longitudinal follow-up into later adolescence would be needed to confirm this trend in our population.

An important methodological consideration is the marked difference between our findings and those reported from India's Comprehensive National Nutrition Survey (CNNS). Vasudevan et al. reported hypertension prevalences of 25-35% among Indian adolescents using nationally representative data [21]. This discrepancy is best explained by differences in classification criteria, measurement technology, and study age range rather than any underlying difference in true BP burden. CNNS classified high BP using the 2017 AAP criteria, which apply a flat ≥ 130/80 mmHg cutoff for adolescents aged 13 years and older, whereas we used the percentile-based Fourth Report criteria [17]. CNNS also measured BP using calibrated oscillometric automated devices rather than auscultation with a mercury sphygmomanometer. Its enrolled age range (10-19 years) extended beyond our cohort's (7-15 years) into the older adolescent years when BP-defined hypertension is most prevalent, consistent with the age-related pattern observed in our own data. Repeated measurements reduce misclassification due to transient physiological variation, anxiety, and the white-coat effect, an observation emphasised in recent global meta-analyses [14,19]. Had we used three separate visits as recommended by the Fourth Report [17] for sustained hypertension, prevalence would likely be lower, as shown by Zhang et al., where prevalence reduced from 17.2% to 4.9% after repeated confirmation [22].

The most important finding is a strong graded association between BMI and hypertension. Hypertension prevalence increased from 2.1% among normal-weight children to 12.3% among overweight and 39.1% among obese children, demonstrating a clear dose-response. Similar findings have been consistently reported across Indian studies. Raj et al. demonstrated progressively increasing hypertension prevalence across BMI categories in Kerala [11], while Meena et al. confirmed substantially higher pooled prevalence among obese than normal-weight children [13]. Comparable gradients were reported internationally, with both Song et al. and Ruan et al. demonstrating progressively increasing prevalence from normal-weight to overweight and obese children [14,19].

Multivariable analysis further strengthens the association. After adjustment, overweight children had approximately threefold higher odds, and obese children had more than eightfold higher odds compared with normal BMI. This effect size closely corresponds with Zhang et al., who reported adjusted ORs of 8.6-14.0 among obese Chinese children following repeated confirmatory measurements [22]. Xu et al. also demonstrated progressively increased odds among overweight and obese Chinese children, although the magnitude was lower than observed in our cohort [23]. Together with evidence from Indian and global meta-analyses [13,14,19], these findings support excess adiposity as a consistent and potentially modifiable factor associated with childhood hypertension across diverse populations.

Beyond adiposity, parental history of hypertension remained associated, consistent with Buch et al. [10]. Male sex and mixed dietary pattern also retained significant associations after adjustment. In contrast, physical activity <30 min/day and frequent junk food consumption were strongly associated in unadjusted analyses but not significant after adjustment for BMI and other covariates. Attenuation suggests crude relationships may partly reflect differences in adiposity or other correlated characteristics. However, cross-sectional design does not permit formal conclusions regarding mediation or causal pathways. Moreover, broad classification of dietary pattern and frequency-based assessment may not capture total energy intake, sodium consumption, or overall dietary quality.

School type and socioeconomic status showed a similar pattern of attenuation after multivariable adjustment. Private-school attendance was associated with more than two-fold higher crude odds but not independently associated after accounting for BMI and other covariates. Lower socioeconomic status likewise was associated in unadjusted analysis but not in the final model. Indian studies have reported considerable variation across socioeconomic settings. Goel et al. reported relatively high prevalence among lower-middle socioeconomic communities in urban Mumbai [24], whereas Rajinikanth et al. observed lower prevalence among private-school adolescents in Chennai despite confirming association between obesity and hypertension [25]. James et al. similarly reported lower prevalence among rural schoolchildren while identifying obesity as an important correlate [26]. Collectively, findings indicate that associations between socioeconomic context, school environment, and hypertension are heterogeneous and may be partly explained by differences in adiposity and associated behaviours.

Strengths and limitations

Strengths include inclusion of both government and private schools capturing socioeconomic diversity, standardized anthropometry with calibration, mean of three BP readings reducing single-reading misclassification, and use of a multivariable model. This is among the few studies from south-central India reporting BMI, diet, socioeconomic status, and family history together.

Several limitations should be acknowledged. First, data were collected in 2014-2015 and analysed later; lifestyle patterns, dietary habits, and obesity prevalence may have changed. However, close alignment with Meena et al.'s [13] pooled estimate supports continued relevance, though current prevalence may be higher. Second, BP was assessed on a single day, rather than three separate occasions as recommended for sustained hypertension, and without ambulatory BP monitoring; this may overestimate hypertension and misclassify white-coat or masked hypertension. Third, we used Fourth Report 2004 criteria [17], and the use of the American Academy of Pediatrics 2017 Clinical Practice Guideline would likely alter prevalence estimates. Fourth, socioeconomic status was classified per the modified Kuppuswamy 2007 scale, which may misclassify for 2014-2015 due to inflation. Fifth, dietary pattern, junk food consumption, physical activity, and parental history were self-reported and may be affected by recall and reporting bias. Sixth, no detailed measurements of dietary energy intake, sodium consumption, central adiposity (waist circumference), pubertal staging by Tanner, or biochemical cardiometabolic markers, which may contribute to residual confounding. Seventh, cross-sectional design precludes causal inference. Finally, participnts were recruited from selected schools within a single metropolitan region, limiting generalizability to rural populations and other regions of India. Convenience sampling may introduce sampling bias. Possible design effects and clustering need to be addressed by cluster-adjusted modelling.

## Conclusions

In this school-based cross-sectional study in Hyderabad, conducted in 2014-2015, hypertension and prehypertension were relatively common among children aged 7-15 years, and the prevalence of prehypertension and hypertension rose steadily with age. BMI showed a strong graded association with hypertension, with obesity associated with substantially higher odds after adjustment. Parental history of hypertension, male sex, and mixed dietary pattern were also independently associated with hypertension. Because BP was measured on a single day and the study was restricted to selected schools in one metropolitan region, these findings should be interpreted as associations rather than causal relationships. Multicenter studies using repeated-visit BP measurements and contemporary pediatric BP criteria are warranted.

## References

[1] Sorof JM, Lai D, Turner J, Poffenbarger T, Portman RJ (2004). Overweight, ethnicity, and the prevalence of hypertension in school-aged children. Pediatrics.

[2] Muntner P, He J, Cutler JA, Wildman RP, Whelton PK (2004). Trends in blood pressure among children and adolescents. JAMA.

[3] Bagga A, Jain R, Vijayakumar M, Kanitkar M, Ali U (2007). Evaluation and management of hypertension. Indian Pediatr.

[4] Jafar TH, Islam M, Poulter N, Hatcher J, Schmid CH, Levey AS, Chaturvedi N (2005). Children in South Asia have higher body mass-adjusted blood pressure levels than white children in the United States: a comparative study. Circulation.

[5] Bhave S, Bavdekar A, Otiv M (2004). IAP National Task Force for childhood prevention of adult diseases: childhood obesity. Indian Pediatr.

[6] Bhardwaj S, Misra A, Khurana L, Gulati S, Shah P, Vikram NK (2008). Childhood obesity in Asian Indians: a burgeoning cause of insulin resistance, diabetes and sub-clinical inflammation. Asia Pac J Clin Nutr.

[7] Kotchen TA (2010). Obesity-related hypertension: epidemiology, pathophysiology, and clinical management. Am J Hypertens.

[8] Mohan B, Kumar N, Aslam N, Rangbulla A, Kumbkarni S, Sood NK, Wander GS (2004). Prevalence of sustained hypertension and obesity in urban and rural school going children in Ludhiana. Indian Heart J.

[9] Sharma A, Grover N, Kaushik S, Bhardwaj R, Sankhyan N (2010). Prevalence of hypertension among schoolchildren in Shimla. Indian Pediatr.

[10] Buch N, Goyal JP, Kumar N, Parmar I, Shah VB, Charan J (2011). Prevalence of hypertension in school going children of Surat city, Western India. J Cardiovasc Dis Res.

[11] Raj M, Sundaram KR, Paul M, Deepa AS, Kumar RK (2007). Obesity in Indian children: time trends and relationship with hypertension. Natl Med J India.

[12] Bagudai S, Nanda P, Kodidala SR (2014). Prevalence of obesity and hypertension in adolescent school-going children of Berhampur, Odisha, India. Int J Physiother Res.

[13] Meena J, Singh M, Agarwal A, Chauhan A, Jaiswal N (2021). Prevalence of hypertension among children and adolescents in India: a systematic review and meta-analysis. Indian J Pediatr.

[14] Ruan X, Zhu A, Wang T (2025). Global prevalence of hypertension in children and adolescents younger than 19 years: a systematic review and meta-analysis. JAMA Pediatr.

[15] Kumar N, Shekhar C, Kumar P, Kundu AS (2007). Kuppuswamy's socioeconomic status scale-updating for 2007. Indian J Pediatr.

[16] Khadilkar VV, Khadilkar AV, Choudhury P, Agarwal KN, Ugra D, Shah NK (2007). IAP growth monitoring guidelines for children from birth to 18 years. Indian Pediatr.

[17] Falkner B, Daniels SR, Flynn JT (1996). The Fourth Report on the Diagnosis, Evaluation, and Treatment of High Blood Pressure in Children and Adolescents. Pediatrics.

[18] Daniel RA, Haldar P, Prasad M, Kant S, Krishnan A, Gupta SK, Kumar R (2020). Prevalence of hypertension among adolescents (10-19 years) in India: a systematic review and meta-analysis of cross-sectional studies. PLoS One.

[19] Song P, Zhang Y, Yu J, Zha M, Zhu Y, Rahimi K, Rudan I (2019). Global prevalence of hypertension in children: a systematic review and meta-analysis. JAMA Pediatr.

[20] Zhou J, Shan S, Wu J (2026). Global prevalence of hypertension among children and adolescents aged 19 years or younger: an updated systematic review and meta-analysis. Lancet Child Adolesc Health.

[21] Vasudevan A, Thomas T, Kurpad A, Sachdev HS (2022). Prevalence of and factors associated with high blood pressure among adolescents in India. JAMA Netw Open.

[22] Zhang Q, Yang L, Zhang Y, Zhao M, Liang Y, Xi B (2019). Hypertension prevalence based on three separate visits and its association with obesity among Chinese children and adolescents. Front Pediatr.

[23] Xu H, Hu X, Zhang Q (2011). The association of hypertension with obesity and metabolic abnormalities among Chinese children. Int J Hypertens.

[24] Goel A, Goel P (2024). Investigating the relationship between hypertension and obesity in schoolchildren from lower-middle socioeconomic strata in urban Mumbai. Cureus.

[25] Rajinikanth SB, Sujatha U, Yadav S (2023). Prevalence of obesity and its relationship with hypertension among school-going adolescents aged 12-16 years. Cureus.

[26] James BC, Venkateswaran A, Agneeswaran A, Premkumar B, Sanghavi B (2023). The prevalence of elevated blood pressure and its association with obesity in children aged 6-13 years in rural India: a cross-sectional study. Cureus.

